# Sublethal pesticide exposure influences behaviour, but not condition in a widespread Australian lizard

**DOI:** 10.1093/conphys/coac024

**Published:** 2022-04-24

**Authors:** Isabella Contador-Kelsall, Kimberly Maute, Paul Story, Grant C Hose, Kristine French

**Affiliations:** School of Earth, Atmospheric and Life Sciences, University of Wollongong, Northfields Ave, Wollongong, 2522 New South Wales, Australia; School of Earth, Atmospheric and Life Sciences, University of Wollongong, Northfields Ave, Wollongong, 2522 New South Wales, Australia; Australian Plague Locust Commission, Unit 7, 50 Collie St, Fyshwick ACT 2609 Australian Capital Territory, Australia; Faculty of Science & Engineering, 14 Sir Christopher Ondaatje Ave, Macquarie University, Sydney, 2109 New South Wales, Australia; School of Earth, Atmospheric and Life Sciences, University of Wollongong, Northfields Ave, Wollongong, 2522 New South Wales, Australia

**Keywords:** pesticide, lizard, ecotoxicology, body condition, arid, Activity

## Abstract

Assessment of non-target impacts of pesticides used widely in agriculture and pest management rarely considers reptiles. Despite their integral role in all ecosystems, particularly arid ecosystems, reptiles are not included in risk assessments. Two pesticides used in agricultural pest management are fipronil and fenitrothion. Here, we used a field-based BACI design experiment in semi-arid Australia to investigate the impact of these pesticides on basic physiological and behavioural parameters of a common arid-zone lizard species, *Pogona vitticeps*. Fipronil and fenitrothion were applied at ecologically relevant doses via oral gavage. Before and after dosing, blood, physical activity and body condition parameters were assessed. We found that temperature significantly influenced lizard activity in the morning period of movement; however, fipronil-treated individuals moved at least 49% less than fenitrothion-treated and control lizards from 7 days after dosing through to the end of the experiment. Physiological measures did not change significantly before or after exposure to both pesticides; however, other indicators showed evidence of exposure, which remained for the entirety of our monitoring period. On average, cholinesterase inhibition was still >30% compared with control lizards at the end of 4 weeks, and fipronil sulfone blood residues remained at 0.219 μg/ml. Our study provides novel insights into the impacts that common pesticides have on widespread lizard species. We show that an ecologically relevant low dose of fipronil alters the behaviour of *P. vitticeps*, which has the potential to impact longer-term survivability. Persistence of both pesticides in the blood of all treatment lizards throughout the experiment indicates they are unable to clear these toxins within a month of being exposed. This may be significant for compounding exposure and latent toxicity. These findings highlight the susceptibility that reptiles have to a selection of common pesticides and the inherent need for higher prominence in wildlife ecotoxicological research.

## Teaser

Reptiles are overlooked in ecotoxicological research. We assessed the impacts of two commonly used pesticides, fipronil and fenitrothion, on a widespread Australian lizard, using ecologically relevant exposure. Fipronil reduced activity by ≥49% but did not impact physiological measures. Fenitrothion exposure did not result in detectible changes to behaviour or physiology.

## Introduction

Pesticide use is a prominent and ongoing practice in agriculture. Insecticides are used globally for protection of plants, animals and their products (Simon-Delso *et al.,* 2015). The safe and sustainable use of pesticides is informed by ecological risk assessments, which are formulated based on existing knowledge and research (Weir *et al.,* 2010; Lopez-Antia *et al.,* 2016). In general, risk assessments globally only require evidence that pesticides do not pose a high-enough risk to mammals, fish, aquatic invertebrates and birds, but there is no requirement regarding reptiles (Amaral *et al.,* 2012b). Relative to most vertebrates, there remains a dearth of toxicity data for herpetofauna (Story and Cox, 2001). The majority of toxicity data are for mammals (38%) and birds (31%), whereas reptiles are represented in only 2% of studies (Gibbons *et al.,* 2015), and reptile studies account for only 0.8% of ecotoxicology citations (Sparling *et al.,* 2010). Whether this is a consequence of a lack of incentive to study, or a lack of available data, reptiles are typically not considered in pesticide use registrations worldwide, leaving them severely underrepresented in current risk assessments and ecotoxicological studies (Campbell and Campbell, 2002; Amaral *et al.,* 2012b; Gardner and Oberdorster, 2016).

Reptiles, more specifically lizards, play significant roles within ecosystems, and their biology and life history suggest they are sound bioindicators of environmental contaminants. Lizards tend to have generalist omnivorous diets, are long-lived, inhabit a range of habitats pertaining to a large geographic distribution (Fossi *et al.,* 1995) and tend to display site fidelity (Crain and Guillette Jr, 1998; Amaral *et al.,* 2012b; Lambert, 2005; Nasri *et al.,* 2020). The ecological role of lizards is more crucial in arid ecosystems given the high lizard abundance and their importance within the food chain, as omnivores and secondary consumers (Pianka, 1986). Reptiles are predicted to have a high susceptibility to bioaccumulation given their ectotherm metabolism, generalist diet, placement in the food chain (Lambert, 1997; Campbell and Campbell, 2002) and site fidelity (if a site is contaminated). Therefore, lizards are candidates for significant exposure and should be integral inclusions in future ecological and wildlife ecotoxicological research (Maute *et al.,* 2015; Nasri *et al.,* 2020).

Aerial application of insecticides is a key element of agricultural pest control strategies globally (Simon-Delso *et al.,* 2015). Pesticides can be applied to large areas by aircraft spraying ultra-low volume formulations (Story and Cox, 2001; Sinervo *et al.,* 2010). In the arid zone of Australia, fenitrothion (O,O-dimethyl-O-(3-methyl-4-nitrophenol)-phosphorothioate, CAS 122-14-5) and fipronil (5-amino-3-cyano-1-(2,6-dichloro-4-trifluoromethylphenyl)-4-trifluoromethylsulfinyl pyrazole, CAS 120068-37-3) are applied aerially for locust control (Story *et al.,* 2013; Maute *et al.,* 2017; APLC, 2020). Fenitrothion is a broad-spectrum organophosphate insecticide widely used in agricultural pest control (Story *et al.,* 2021). It acts by inhibiting cholinesterase activity, a neurotransmitter that is essential for normal cholinergic innervation in both the central nervous system (CNS) and peripheral nervous system (Story and Cox, 2001). Cholinergic innervation is an essential and ubiquitous function in vertebrates, and, in turn, suppression of acetylcholinesterase (AChE) activity can have far-reaching effects on a range of physiological parameters including thermoregulation, endocrine function, metabolism, locomotion and feeding behaviours (Story and Cox, 2001; Weir *et al.,* 2010; Story *et al.,* 2021). Fipronil is a phenyl pyrazole insecticide that is extensively used as a veterinary and agricultural insecticide (Tingle *et al.,* 2003). Fipronil disrupts the action of gamma-amino butyric acid (GABA), an inhibitory neurotransmitter necessary for the function of the CNS. Fipronil acts by blocking critical chloride channels through binding to post-synaptic receptors that are essential for GABA innervation (Hainzl and Casida, 1996; Narahashi, 2010; Story *et al.,* 2021).

Previous laboratory experiments have shown lizard sensitivity to fipronil (Peveling and Demba, 2003), as well as a field-based experiment showing the significant reduction in relative abundance of two lizard species populations in Madagascar (Peveling *et al.,* 2003). While a single field study in Australia has suggested that reptile abundance and community composition was not impacted by fipronil application for locust control (Maute *et al.,* 2015), Bain *et al.* (2004) and Van de Weyer (2017, unpublished manuscript) are the only studies where the direct impact of a pesticide (fenitrothion or fipronil) was investigated in an Australian reptile at the individual level in the laboratory.

Aerial spraying of insecticides translates into oral exposure for omnivorous reptiles, via consumption of vegetable matter and insect prey. Using oral gavage to assess the impact of insecticides accounts for all potential exposure likely from a spray event. Investigating physiological and behavioural markers of exposure such as changes in activity and body condition parameters can be extremely informative for assessing overall impact of pesticide exposure on the body (Green, 2001; Amaral *et al.,* 2012a; Amaral *et al.,* 2012b; Dantzer *et al.,* 2014; Abrahms *et al.,* 2016). The objective of this study was to investigate the impact of sublethal oral exposure of two pesticides used in agricultural pest management on a common native Australian lizard, the central bearded dragon (*Pogona vitticeps*, Ahl 1926) in a field setting. Body condition, daily activity measures and biomarkers of exposure were measured before and after ecologically relevant pesticide exposure treatments. We predicted that both fenitrothion and fipronil might alter these measures, and results would suggest whether oral exposure to pesticides changes physiology or behaviour in ways that would influence longer-term survival or reproduction.

## Materials and methods

### Study site and species

The study was conducted at Nombinnie Nature Reserve (33°04′25.34′′S, 145°47′28.39″E) from mid-January until mid-March 2019. Nombinnie Nature Reserve consists of 70 000 ha in central NSW encompassing habitats from three biophysical regions (Cobar Peneplain, Darling Depression and Southern Riverine Plain) and containing the largest remaining stretch of Mallee woodland in NSW (NPWS, 1996). Total rainfall for the spring–summer period (September–March) in 2017/2018 was 165.5 mm, whereas total spring–summer rainfall for 2018/2019 was 220.4 mm (Meteorology, 2021b). Historically (1885–2009), mean spring–summer rainfall was 224.7 mm (Meteorology, 2021a). Mean monthly temperatures from January to March in 2019 were 32.5°C, 25.6°C and 22.7°C, respectively, with the highest temperature recorded in January 2019 at 46.9°C. Temperature was recorded via the closest automatic weather station (Euabalong Mount Hope, station ID 049136) run by the Australian Bureau of Meteorology. *Pogona vitticeps* is a common arid-zone agamid that has a wide habitat distribution across arid and semi-arid Australia including dry sclerophyll forests, eucalypt and cypress pine woodlands, *Acacia* scrubs and various desert habitats (Rej and Joyner, 2018). *Pogona vitticeps* are omnivorous, semi-arboreal, medium-bodied terrestrial lizard (Cogger, 2018).

### Animal capture, handling and tracking

*Pogona vitticeps* were captured opportunistically during visual scanning surveys via car or walking. Lizards were captured, sampled and released between 17 days and 1 day pre-exposure to pesticide, from 19 January to 4February depending on the individual. A GPS location was recorded on a mobile device in order to return the individual to the exact location once sampling was complete. Upon capture, individuals were morphologically sexed by hemipenal inspection and snout-to-vent length (SVL) and mass (g) were measured to determine scaled body mass indices (SBMI) (Peig and Green, 2009). An initial blood sample of ~200 μl was taken from the caudal tail vein collecting whole blood for haemoglobin (Hb) measurement, haematocrit tubes for plasma collection and blood spot cards (PerkinElmer 226 Five Spot cards). The entire sampling took less than 30 min per lizard. Once this was complete, they were returned immediately to the exact location captured. Hb was measured using a Hemocue HB 201 (Abgleholm, Sweden) immediately after the blood sample was taken. Blood spot cards were frozen at −20°C while in the field and transferred to −80°C at the conclusion of the field study. Haematocrit tubes were processed in the laboratory on the day of collection; blood was centrifuged at 14000 RCF for 2.5 min (Hematospin 1300, Hawksley and Sons Ltd) and plasma was collected from the centrifuged haematocrit tube and stored as above.

A HOBO accelerometer (HOBO® Pendant G, Onset Computer Corporation) and Sirtrack VHF tag (Sirtrack Ltd, Havelock North, New Zealand) were attached to each lizard on the dorsal anterior side of the tail. VHF tags ranged from 4 to 13 g and accelerometers were ~4 g, total device weight falling within 5–7% of total body mass of each lizard. This was fastened using a small amount of super glue and brown surgical tape for camouflage. Accelerometers were set to record X and Y values in g-force every 30 s during daylight, as pilot data (Bernich *et al.* 2022, in press) suggested animals were rarely active at night. Individual lizards were assigned a four-digit combination code of three colours at initial capture. Paint markers were used under the front and hind legs, hidden from plain sight, according to assigned ID. During the final sample session, individuals were microchipped subcutaneously for permanent identification (Trovan Unique Midichip All-in-One).

Lizards were sampled at pre-determined sampling sessions following initial capture and dosing. Dosing took place over 2 days (4–5 February), with each pesticide allocated to a single day. These sample sessions were post-24 hours, post-7 days, post-14 days and post-28 days after pesticide dosing. At each sample session we located individuals using their allocated VHF tag. Captured animals had a blood sample (200 μl) taken, as well as SVL and mass. Accelerometers were removed to download all data since previous capture and then reattached. At the final sample session on 3–6 March depending on the individual (post-28 days) accelerometers and VHF tags were permanently removed. All samples and protocols were performed and collected under animal ethics approval through the University of Wollongong under AE17/19 and permitted through NSW National Parks and Wildlife Service Scientific License (SL100109).

### Pesticide dosing

Lizards were randomly allocated to one of three dosing treatments after initial capture: with fenitrothion (*n* = 5), fipronil (*n* = 5) or technical grade corn oil (control, *n* = 6). Both fenitrothion and fipronil were suspended in corn oil, similar to the formulations used in aerial spraying. As fipronil was a powder it was first dissolved in a minimum amount of acetone (acetone solubility = 54.6 g/100 ml) (Tingle *et al.,* 2003; Kitulagodage *et al.,* 2011a), before suspension in corn oil. Pesticide dose was calculated based on previous literature and pilot data incorporating maximum feeding rates and pesticide residue levels found on natural prey and vegetation after locust control operations in Australia (Szabo, 2005; Story *et al.,* 2013), with the aim to administer an ecologically relevant dose. Mimicking the average diet of an adult lizard (70% vegetation and 30% insects) we applied this knowledge to known residue levels of both pesticides on vegetation and locusts. Fipronil residue levels are between 1.4 and 5.6 μg/g on vegetation (1.4 μg/g was used in this experiment) and 0.124 μg/g on locusts with residues persisting for 5 days after application (Szabo, 2005; Story *et al.,* 2021), where fenitrothion residue levels are 62.0 μg/g on vegetation and 39.8 μg/g on locusts with residues persisting for 3 days after application (Story *et al.,* 2013). Fenitrothion lizards received 16.60 mg/kg of fenitrothion and fipronil lizards received 0.51 mg/kg of fipronil in a single dose, based on the degradation pathway and residue persistence of each pesticide and a gorge feeding event (Szabo, 2005; Wilson and Swan, 2013). While a repeated exposure dosage was considered, to match the degradation pathway of each pesticide and the feeding patterns of lizards, it was not a feasible option. Although potentially a more realistic dose scenario, the multiple consecutive captures and gavage protocol would case high stress to all individuals and likely interfere with pesticide intake and viability of our study. As a result, we chose an equally appropriate single dose that represents the most ecologically relevant intake of pesticide that could be realistically studied. A stock solution of each pesticide was formulated, and further dosage was then calculated based on body mass (g) recorded at the first sampling session.

The delivery of pesticides and/or corn oil was via oral gavage using a Covidien Kendall™ plastic feeding tube (6 × 410 mm). The solution was slowly dispensed using a sterile 5-ml syringe and then flushed with 1 ml of pure corn oil.

### Biomarkers of exposure

#### Fipronil

All samples were prepared according to Raju *et al.* (2016). A single punch was taken from each dry blood spot card (DBS) and placed in an Eppendorf tube containing 100 μl of MilliQ water. The Eppendorf tube was centrifuged ensuring the DBS disc was completely immersed, followed by 5 min sonication. A 300-μl aliquot of acetonitrile was added to each sample followed by vortexing (2 min) and sonication (5 min). A supernatant (200 μl) was collected after centrifugation at 10000 rpm for 10 min. All preparation steps were performed at 4°C.

All samples were analysed using an Agilent 6490 Triple Quad Liquid chromatography–mass spectrometry. All standards for fipronil parent and metabolite compounds (fipronil, fipronil desulfinyl, fipronil sulfide and fipronil sulfone) were purchased from Sigma-Aldrich. Typical R^2^ for each calibration curve was higher than 0.996. A positive control (0.01 μg/ml fipronil) and negative control (acetonitrile) were run every five samples to ensure no contamination. All compounds were run in negative mode, clearly defined and confirmed by their most abundant product ions at optimized collision energies.

#### Fenitrothion

All plasma samples were measured using the Ellman assay (Ellman *et al.,* 1961), as modified by Gard and Hooper (1993). AChE and total plasma cholinesterase (ChE) activity suppression were measured in plasma samples, with butrylcholinesterase (BChE) activity calculated as the difference between TChE and AChE. Difference from control samples indicated AChE suppression and, consequently, exposure to fenitrothion. All assay reagents were purchased from Sigma-Aldrich. Mouse serum (Sigma-Aldrich, Australia) was used as a between-assay standard and samples without the addition of enzyme were used as blanks. All samples were run at an optimal dilution ratio of 1:5 in duplicates or triplicates dependent on amount of plasma available. Detailed methodology of the assay can be found in the Buttemer *et al.* (2008) and the appendix.

### Activity measures

Activity measurements were measured on an X- and Y-axis in g-force at 30 s intervals. We removed data surrounding sampling sessions (10 min before capture and 20 min after release), to remove any confounding impacts of capture on lizard activity measures. All other data were grouped into 10-min intervals of potential activity and then into daily totals of minutes of activity. We categorized a threshold for movement or no movement within the 10-min intervals as 0.0199 g^2^, the smallest amount of variance that represented animal movement, following Bernich et al. (2022, in press).

Activity data were split into two daily sessions. The morning activity period was defined as 06:00–12:00 (AM) and the afternoon activity period was defined 13:00–19:00 (PM), based on previous field observations. In each session, a sample of three 24-h periods were chosen surrounding the sample time (before or after the date depending on session) for which mean total activity data in minutes was calculated, based on adding the 10-min intervals. Time of first movement was recorded as the first movement of the AM period with a single time value per lizard for each sample session. These datapoints were treated the same way, analysing only three 24-h periods for each sample session.

**Table 1:** Best-fit repeated measures linear model detailing the relationship between scaled SBMI and Hb separately over time and across treatments

Response variable	Explanatory variable	Numerator df	Denominator df	*F*	*P*	AICc
	Time	4	43.04	3.493	**0.015**	
SBMI	Treatment	2	11.02	1.397	0.288	515.960
	Time^*^treatment	8	43.03	0.788	0.616	
	Time	4	39.26	3.547	**0.015**	
Hb	Treatment	2	10.99	1.438	0.279	374.409
	Time^*^treatment	8	39.24	0.346	0.942	

*Notes:* Significant effects are printed in bold. Degrees of freedom (df), *F* statistic (*F*), *P*-value (*P*) and Hurvich and Tsai’s Criterion (AICc) are a result of a restricted maximum likelihood model, where individuals are repeatedly sampled over time.

### Statistical analysis

Assumptions of normality for each dataset were confirmed prior to analysis. To test the effect of pesticides described below and assuming a linear-no-threshold relationship, we used backward elimination to select best-fit repeated measures linear models and multiple linear regression models using IBM SPSS Statistics (Ver. 26). In general, we started with a full model including all relevant explanatory variables (dependent on the model response variable) and their interactions and sequentially removed fixed effect variables that had the highest *P*-values. Model selection was also based on guidance from AICc values. All models were fit using ReML and assessed by *F*-tests set at type III sum of squares. Tukey HSD *post hoc* tests were run in order to pinpoint the direction of significant differences among means for significant response variables.

To test the effect of time and treatment on Hb and SBMI we ran best-fit repeated measures linear models, where time and temperature were fixed effects with their interaction term. Sex was initially included in the model but was eliminated due to lack of usefulness. Treatment and lizard ID were specified as subjects. To test whether time, treatment or temperature influenced activity measures (as mean minutes moved AM or PM and time of first movement) we ran best-fit repeated measures linear models. Explanatory variables SBMI, Hb and sex were eliminated from the model via the method described above. Treatment and lizard ID were specified as subjects and time was specified as repeated, with the repeated covariance type defined as compound symmetry. Explanatory variables included temperature and one interaction term between time and treatment. A marginal R^2^ (Nakagawa and Schielzeth, 2013) was obtained in order to explain significance of temperature in mean minutes moved in the morning period (AM). A single best-fit repeated measures linear model was run to test the effect of time and treatment on total plasma ChE and AChE in the blood. Mean minutes moved, SBMI and Hb were eliminated from the full model. Treatment and lizard ID were specified as subjects and time was specified as repeated. Explanatory variables included one interaction term between time and treatment.

We ran four best-fit multiple linear regression models to test the influence of fipronil sulfone, lizard ID, time, treatment and temperature on mean minutes moved AM or PM. Time was removed from fipronil grouped models as it was highly related to fipronil sulfone levels over time. A Pearson’s correlation matrix was run for temperature (AM) and fipronil sulfone to assess collinearity based on visual interpretation (Fig. 2) prior to regression analysis. A Pearson correlation coefficient of <0.5 (see Appendix Table A4) suggested the two explanatory variables were not significantly correlated and could be used in analysis (Zuur *et al.,* 2009). Fipronil grouped models did not include treatment as control individuals have no fipronil sulfone in their blood. In contrast, fenitrothion grouped models required the inclusion of treatment as an explanatory variable given that ChE is present in healthy (control) individuals at all times. For three out of the six control lizards the total plasma ChE and AChE values were missing for time point 1. For these animals we used time point 2 as time point 1 in order to preserve the dataset. We tested both the full (six lizards in total) and a reduced dataset (three lizards in total) and results were similar and interpreted in the same way.

**Figure 1:**
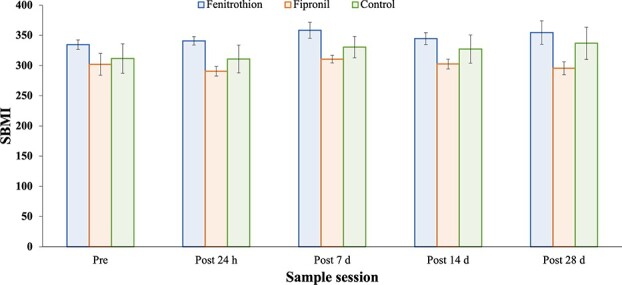
SBMI of *P. vitticeps* in all treatments (fipronil, fenitrothion, control) at five time points. SBMI data are shown as means with ± SE (4–6 animals per treatment).

**Table 2:** Summary of best-fit repeated measures linear models detailing the relationship between activity levels and time, treatment and temperature

Response variable	Explanatory variable	Numerator df	Denominator df	*F*	*P*	AICc
Mean mins moved AM	Time	4	43.00	2.509	0.056	480.933
	Treatment	2	9.54	3.143	0.089	
	Time^*^treatment	8	40.09	1.367	0.240	
	TemperatureAM	1	34.79	7.877	**0.008**	
Mean mins moved PM	Time	4	40.41	3.080	**0.026**	512.452
	Treatment	2	10.46	0.680	0.528	
	Time^*^treatment	8	39.06	0.354	0.938	
	TemperaturePM	1	48.84	0.163	0.688	
Time of first movement	Time	4	41.98	5.909	**0.001**	1003.574
	Treatment	2	10.66	3.273	0.078	
	Time^*^treatment	8	40.09	1.614	0.151	
	TemperatureAM	1	49.92	3.172	0.081	

*Notes:* Significant effects are printed in bold. Degrees of freedom (df), *F* statistic (*F*), *P*-value (*P*) and Hurvich and Tsai’s Criterion (AICc) are a result of a restricted maximum likelihood model, where individuals are repeatedly sampled over time. AM is defined as 6 am—12 pm and PM is defined as 1 pm—7 pm.

## Results

### Body condition

A total of 16 *P. vitticeps* were measured over the experimental period enabling 4–6 individuals to be allocated per treatment. Individuals varied in their size and physiological parameters. Mean (± SD) SVL among lizards was 198.4 ± 18.5 mm and mean Hb concentration was 92.2 ± 12.5 g/l on initial capture. Mean Hb concentrations fluctuated the most for control individuals with a 12% decrease from pre-dose to post-28 days dose, with fenitrothion individuals showing a 7% decrease and fipronil individuals showing no change, respectively. Initial mean mass of all animals was 260.9 ± 69.7 g with a mean SBMI of 313.1 ± 13.7.

There was no significant effect of either pesticide on the physiology of *P. vitticeps*. There were no significant effects of pesticide treatments on any of the variables relating to body condition (Hb, SBMI). The assumption of sphericity (Maulchey’s Test of Sphericity) was met for both response variables. Regardless of treatment, there was a significant effect of time on SBMI (Table 1; Fig. 1), which increased by up to 6% over the experimental period. However, Tukey HSD was not able to distinguish where the differences lay. The largest difference of 6% was between post-24 h and post-7 days (see Appendix). Hb concentration also significantly changed over time for all individuals regardless of treatment (Table 1), decreasing by 6% over the experimental period. A Tukey HSD was not able to delineate where differences lay; however, the largest difference was a 7% decrease between post-24 h and post-7 days (see Appendix).

**Figure 2:**
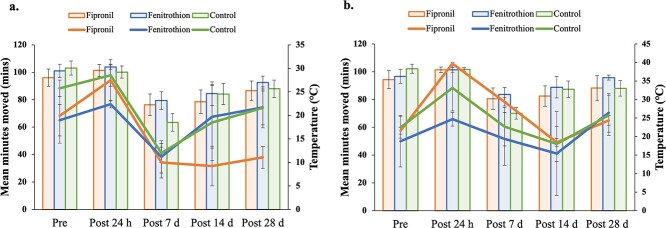
Mean minutes moved by *P. vitticeps* between 6 am–12 pm (a) and 1–7 PM (b) for all treatments against mean temperature at five time points. Column graph denotes temperature and line graph denotes activity, with data displayed as means with ± SE (4–6 animals per treatment).

**Figure 3:**
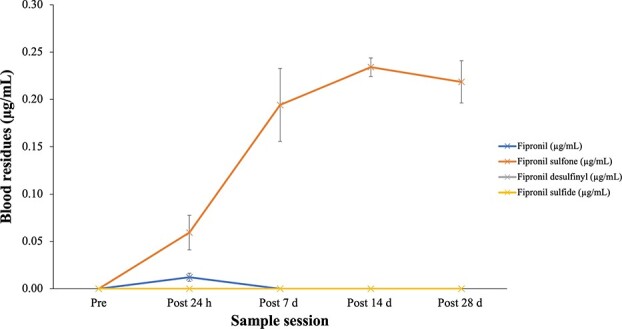
Fipronil (μg/ml) and its metabolites (fipronil sulfone, fipronil sulfide, fipronil desulfinyl) in fipronil-dosed *P. vitticeps* (*n* = 4) at five time points. Data is displayed as means with ± SE.

### Activity

We recorded a minimum of 80 640 activity data points for each lizard over the experimental period; the number of data points collected for each animal was dependent on date of initial capture. Neither time nor treatment had a significant effect on mean minutes moved in the morning period; however, mean morning temperature had a significant effect on this measure (Table 2; Fig. 2a). The marginal R^2^ value for this model was 40.4%, with temperature explaining 30.8% of variation. This relationship with morning temperature is shown most strongly between post-24 h and post-7 days where mean temperature decreases 8.4°C coupled with a decrease of 58% in mean activity during the morning period (Fig. 2a). Mean temperature continued to increase by 4.7°C between post-7 days and post-28 days with an increase in mean morning activity by 39% (Fig. 2a). While there was no significant treatment effect on mean minutes moved in the morning, there was a clear trend in which fipronil-treated individuals were at a minimum 49% less active than fenitrothion and control individuals from 14–28 days post-dose (Fig. 2a). For mean minutes moved during the afternoon period, time had a significant effect (Figure 2b; Table 2). The most notable change in mean afternoon activity was seen between pre- and post-24 h where activity increased by 55% (Fig. 2b). Mean minutes moved in the afternoon was not significantly influenced by treatment or temperature (Table 2). Similarly, treatment and temperature had no significant effect on time of first movement, although time did have a significant effect (Table 2). A Tukey HSD picked up multiple significant differences between time points, with the largest difference between post-24 h and post-7 days (*P* < 0.0001) (see Appendix). On average time of first movement was 40% later or 2 h and 56 min later in post-7 days compared with post-24 h.

### Biomarkers of exposure

**Figure 4:**
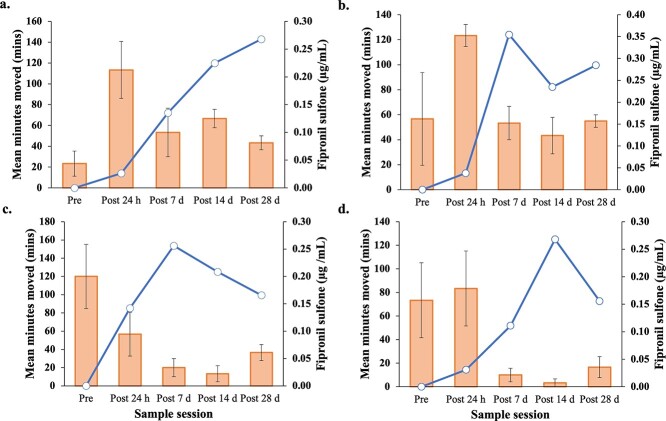
Mean minutes moved between 6 am and 12 pm across five time points plotted against fipronil sulfone (μg/ml) levels in the blood of fipronil-dosed *P. vitticeps*. Mean minutes moved indicated by the column graph (± SE) and fipronil sulfone (μg/ml) levels indicated by the line graph. Individual ID is as follows: a = BPPP, b = PBYY, c = BBPY, d = BYPY.

Fipronil blood residue was measured at 24 h post-dosing in all fipronil-treated individuals (mean, 0.012 μg/mL ± 0.01). Fipronil was not detected in fipronil-treated individuals in any subsequent sampling sessions (post-7 days, pos- 14 days, post-28 days) (Fig. 3). Fipronil sulfone, which is one of the three fipronil metabolites tested, was the only metabolite detected in any of the treatment individuals. Fipronil sulfone significantly increased over the experimental period, remaining high at post-28 days, with only a 7% decrease from post-14 days. An average peak was measured at post-14 days (0.234 μg/ml ± 0.03; Fig. 3); however, on an individual level 50% of fipronil-treated individuals saw a continued increase in fipronil sulfone residue levels at post-28 days (Fig. 4). Fipronil sulfide and fipronil desulfinyl residues were not detected in the blood of any treatment individuals over the entire experimental period (Fig. 3).

Fipronil sulfone levels in the blood of fipronil-treated individuals was associated with a statistically significant decrease the mean minutes moved in the morning period over the duration of the experiment (Table 3; Fig. 4). Mean temperature during the morning period also had a significant positive effect on mean minutes moved (Table 3). Movement of individual fipronil-treated lizards (individual variation) did not significantly influence means minutes moved in the morning period (Table 3). This was the opposite for mean minutes moved in the afternoon period, where individual fipronil-treated lizards significantly influenced activity, more than fipronil sulfone residues or temperature (Table 3). Fipronil sulfone and temperature separately did not significantly influence the activity of fipronil treated individuals in the afternoon (Table 3).

**Table 3:** Summary of best-fit multiple linear regression models detailing the relationship between activity levels and fipronil sulfone (*n* = 4), ChE (*n* = 10) or AChE (*n* = 10) activity levels and temperature

Response variable	Explanatory variable	*t*	*P*	R^2^ (%)
Mean mins moved AM (fipronil group)	Fipronil sulfone	−2.413	**0.028**	61.7
	TemperatureAM	3.411	**0.004**	
	LizardID	1.510	0.151	
Mean mins moved PM (fipronil group)	Fipronil sulfone	−1.167	0.260	32.8
	TemperaturePM	0.853	0.406	
	LizardID	2.344	**0.032**	
Mean mins moved AM (fenitrothion and control groups)	ChE	0.445	0.659	33.1
	TemperatureAM	3.748	**<0.001**	
	LizardID	0.116	0.909	
	Treatment	0.632	0.532	
Mean mins moved AM (fenitrothion and control groups)	AChE	0.489	0.628	32.9
	TemperatureAM	3.720	**<0.001**	
	LizardID	0.110	0.913	
	Treatment	0.603	0.551	
Mean mins moved PM (fenitrothion and control groups)	ChE	1.313	0.199	28.7
	TemperaturePM	1.043	0.305	
	LizardID	3.273	**0.003**	
	Treatment	−2.055	0.048	
Mean mins moved PM (fenitrothion and control groups)	AChE	1.627	0.114	26.0
	TemperaturePM	0.869	0.392	
	LizardID	3.051	**0.005**	
	Treatment	−1.998	0.055	

*Notes:* Significant effects are printed in bold. ChE, AChE, T statistic (t), *P*-value (*P*) and R^2^ are a result of a multiple linear regression. AM is defined as 6 am–12 pm and PM is defined as 1–7 pm.

Total plasma ChE and AChE activity levels in the blood of fenitrothion treated and control individuals were found to have no effect on mean minutes moved in either the morning or afternoon period over time (Table 3). Temperature significantly influenced mean minutes moved in the morning (Table 3) but not in the afternoon period. Minutes moved by individual lizards did not differ significantly in the morning period but did so in the afternoon period for both ChE and AChE (Table 3).

**Figure 5:**
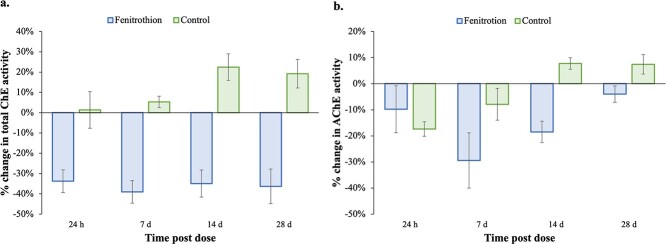
Mean percentage change of ChE activity (μmol substrate/min/ml) (a) and AChE activity (μmol substrate/min/ml) (b) in fenitrothion-dosed and control *P. vitticeps* (4–6 animals per treatment) at four time points after being dosed. Data is displayed as means with ± SE.

Total plasma ChE and AChE activity was measured in fenitrothion and control lizards. The effect of the treatment on ChE levels varied over time, but not for AChE (interaction term, Table 4). Mean overall initial ChE levels were 0.634 ± 0.27 μg/ml, and AChE levels were 0.365 ± 0.13 μg/ml. Total plasma ChE inhibition was consistently above 30% for fenitrothion-treated lizards over the experimental period, where AChE peaked with 29% inhibition at 7 days post-dose, but then increased for the remaining duration of the experiment. Control lizards increased consistently in ChE and AChE activity levels in the blood over the experimental period (Fig. 5).

**Table 4:** Best-fit repeated measures linear model detailing relationship between ChE activity levels and AChE activity levels in the blood across time and treatments

Response variable	Explanatory variable	Numerator df	Denominator df	*F*	*P*	AICc
ChE (μg/ml)	Time	4	22.01	4.08	**0.013**	−27.324
	Treatment	1	7.80	0.08	0.792	
	Time^*^treatment	4	22.01	7.90	**<0.001**	
AChE (μg/ml)	Time	4	22.30	1.35	0.285	−40.942
	Treatment	1	8.26	0.48	0.506	
	Time^*^treatment	4	22.30	1.03	0.413	

*Notes:* Significant effects are printed in bold. ChE, AChE, degrees of freedom (df), *F* statistic (*F*), *P*-value (*P*) and Hurvich and Tsai’s Criterion (AICc) are a result of a restricted maximum likelihood model, where individuals are repeatedly sampled over time.

## Discussion

Lizards dosed with fipronil at ecologically relevant concentrations had lower levels of morning activity compared with lizards in the control and fenitrothion treatment groups. Despite low power resulting in a non-significant full model test, there was a ≥49% decrease in activity levels that was significantly related to fipronil sulfone blood residue levels, suggesting a behavioural shift of biological and ecological significance. This could have long-term consequences for the survival of exposed individuals, as normal foraging, mating and predator evasion behaviours may be impacted. The decrease in activity was first recorded 7 days after pesticide dosing and continued to 28 days post-dose (Fig. 2a), suggesting a major shift in lizard behaviour for an extended period of time.

Other studies also suggest that exposure to pesticides can reduce the activity levels of animals. On a broad scale, Hole *et al.* (2005) and Chamberlain *et al.* (2010) showed that mammals and birds accessing organic farm land had higher levels of activity and density compared with conventional farming systems that regularly use pesticides; however, this was dependent on species. Peveling and Demba (2003) investigated the impact of fipronil on various aspects of Fringe-toed Lizard (*Acanthodactylus dumerili*) biology. The fipronil dose used in the study (30 μg/g) was 60 times greater than was used in our study (0.51 μg/g) and resulted in high levels of moribundity (42–62.5%) and mortality (25–50%) in test subjects. Locomotor and feeding activity was significantly reduced for up to 4 weeks post-treatment in fipronil-dosed lizards (Peveling and Demba, 2003). Our study has responded to their call for a more representative scenario of exposure, such as an ecologically relevant dose, to pesticides in the field. Our use of accelerometers provides a non-biased, more precise and data-rich measurement of activity (Jachowski and Singh, 2015) compared with the methods used by Peveling and Demba (2003), enabling us to confirm reduced activity levels of reptiles in response to fipronil exposure, even with the significantly lower fipronil dose used.

The biologically significant change in activity detected in our study using a low dose of fipronil per body weight suggests that any increase in fipronil dosage could have substantial impact on the health of native reptiles. The decrease in activity was only detected in the morning and not in the afternoon and the consequences of this for the maintenance of normal circadian rhythm, survival and growth in free-living populations needs to be determined. However, we could argue that changes in morning activity are more important than those in the afternoon. Generally, moisture levels are highest during the morning in semi-arid and arid ecosystems (Agam and Berliner, 2006), meaning prey and water availability is also higher compared with afternoon periods, making it a more critical time to forage and move through the environment. Crucial reproductive and foraging behaviours are dictated by activity and the ability to move (Sinervo *et al.,* 2010; Jachowski and Singh, 2015; Walker *et al.,* 2015). Reduced activity would not only result in fewer opportunities to mate and forage but also constrain maintenance and growth in lizards (Sinervo *et al.,* 2010). Sinervo *et al.* (2010) attributed climate-driven decreases in activity time during breeding season as potentially contributing to 72% of all global lizard extinctions since 1975. Thus, essential behaviours that are critical for the survival of individuals and populations are most likely impacted by the application of fipronil and need to be included in future risk assessments.

In vertebrates, the parent compound fipronil has three key metabolites. Fipronil is metabolized through various pathways as follows: via oxidation (carried out by monooxygenases within the cytochrome P450 enzyme family) to form fipronil sulfone, via reduction to form fipronil sulfide and via hydrolysis to form fipronil amide (Peveling and Demba, 2003; Mohamed *et al.,* 2004; Simon-Delso *et al.,* 2015). Fipronil sulfone was the sole metabolite detected in our study and was detected at very high concentrations compared with fipronil, which was cleared within 7 days of dosing. Fipronil sulfone itself is 5–10-fold more potent and more persistent, than its parent compound fipronil, with a 6-fold higher binding ability to GABA receptors in vertebrates (Hainzl *et al.,* 1998; Peveling and Demba, 2003). Therefore, fipronil sulfone has the potential to add synergistically to the overall toxicity of a pesticide formulation in exposed vertebrates (e.g. fish and gallinaceous birds), with the specific response likely being dependent on the detoxification pathway of fipronil in each vertebrate class (Hainzl *et al.,* 1998; Peveling and Demba, 2003). This is very important as sulfone persisted in all fipronil-treated individuals for the duration of the experiment, peaking at 14 days post-dose, although remaining high at 28 days post-dose, which is a similar temporal pattern to that observed in an unpublished laboratory study on the same species (Van de Weyer 2017, unpublished manuscript). Equally important is the fact that for 50% of fipronil-treated individuals, sulfone levels in the blood were still increasing at the end of the experiment. Reptiles are known to have lower levels and activity of mixed function oxygenase systems (namely cytochrome P450), which are crucial for the clearance of toxins, compared with other vertebrates (Schwen and Mannering, 1982; Jewell *et al.,* 1989; Peveling and Demba, 2003). Thus, reptiles are believed to have less efficient enzymatic detoxification systems compared with endotherms, resulting in longer detoxification periods (Hopkins, 2005).

In addition to this, fipronil sulfone is lipophilic, meaning it can accumulate in adipose tissue. In numerous species, sulfone has been the main component recovered from lipid stores (Tingle *et al.,* 2003). Reptiles also have energy conversion efficiencies that are 10 times higher than mammals and birds at the same trophic level (Pough, 1980). Bringing all these concepts together, reptiles rely heavily on stored energy, or fat stores throughout times of biological stress, low food availability and brumation (Bryan *et al.,* 1987; Hopkins, 2005). Thus, at these particular times during a lizard’s life, fipronil sulfone residues would be remobilized in a potential cumulative release of toxins, causing latent toxicity to the individual. In mammal and bird-based studies it has also been found that sulfone residues are higher in adipose tissues as opposed to blood (Tingle *et al.,* 2003; Kitulagodage *et al.,* 2011b). This is alarming given the results of this current study, where sulfone levels in the blood were high and correlated with changes in lizard behaviour.

Interestingly, there seems to be a probable and significant relationship between sulfone levels and activity levels. For all fipronil-treated lizards, peak sulfone levels corresponded with comparable decreases in morning in activity levels, and as sulfone levels decreased over time (due to detoxification or storage), there was an increase in activity levels (Fig. 4). Although temperature plays a critical role in reptilian behaviour and activity, this was accounted for by way of unexposed (control) lizards in our experiment, which did not show reduced activity. The cause of the reduced activity in fipronil-dosed lizards may be a consequence of the animals redirecting energy to detoxification processes instead of morning movement behaviours and doing so for the duration of the experiment. Similar results were seen in the Northern Bobwhite Quail, in which there was a strong positive relationship between sulfone levels and weight loss, along with reduced feeding behaviour and a severe reduction in energy levels (Kitulagodage *et al.,* 2011b). We strongly recommend further research investigating these interactions, with larger sample sizes, longer periods of post-exposure observation and incorporating other measures of change in feeding, activity and metabolism.

In contrast to fipronil, the sublethal dose of fenitrothion administered in this study did not influence animal locomotion; a result consistent with earlier studies undertaken by Bain *et al.* (2004) in a laboratory based study. In the current study, fenitrothion-treated individuals did not show a shift in behaviour during either the morning or afternoon movement period when compared with control animals. Fenitrothion causes the inhibition of cholinesterase activity and impacts on animal movement to differing degrees depending on taxa being studied and the dose administered. Total plasma ChE activity decreased consistently and by over 30% throughout the experimental period, where AChE activity also decreased but peaked at 29% at 7 days post-dose. Thus, despite detection, biomarkers of exposure are not being translated and reflected in activity and body condition measures. A probable cause of this is the ecologically relevant dose used, which was not high enough to inhibit AChE activity to the extent where movement was altered. Our results are highly comparable to those of Bain *et al.* (2004) who exposed *P. vitticeps* to a single low (2 mg/kg) or high (20 mg/kg) dose of fenitrothion via oral gavage. Peak ChE inhibition for low-dose individuals reached 19% at 2 h post-dose, whereas peak (68%) inhibition in high-dose individuals was observed 8 h after dosing, with a prolonged inhibition measured for 21 days. This prolonged inhibition was also detected in AChE activity levels where AChE was at 78 ± 7.8% of pre-dose levels after 21 days (Bain *et al.,* 2004). The earliest we sampled lizards in our study was 24 h post-dose (Fig. 5), yet we also detected prolonged inhibition of ChE and AChE for 28 days post-dose. Bain *et al.* (2004) found no significant impacts on body temperature, standard metabolic rate and feeding rate from either doses, similar to the lack of impact found in activity measures and body condition in our study.

Total plasma ChE was significantly inhibited for all fenitrothion-treated individuals for the duration of the experiment, and AChE significantly inhibited for a minimum of 14 days post-dose, yet activity or body condition were not impacted, similar to Bain *et al.* (2004). Fildes *et al.* (2009) and Story *et al.* (2016) investigated the impact of various doses of fenitrothion on three different species of birds and one marsupial mammal and found that locomotor performance and physiological vigour could be significantly impacted for extended periods of time, ranging from 2 to 10 days post-dose. As seen from current published studies, there are no consistent behavioural or physiological changes when different taxa are exposed to fenitrothion, and here we have also showed no clear impacts on *P. vitticeps*, other than ChE and AChE inhibition. This lack of detectable impacts could simply be because fenitrothion can be efficiently cleared from a reptile’s system with little or no physiological or behavioural impacts, despite there still being 30% ChE inhibition in lizards 28 days post-dose. Molecular-based biomarkers of exposure and stress are a potential avenue to explore this further, informatively complimenting current methods used with the potential to yield beneficial information about oxidative stress, a process known to influence physiological stress levels in animals (Johnstone *et al.,* 2012). Oxidative stress assays are widely used and numerous in choice and would provide an insight to the molecular impacts of such pesticides, potentially significant in both the short and long term.

Ectotherms rely on behaviour to regulate their body temperature. Ambient temperature and external environmental factors are responsible for how active a lizard will be at any given time (Heatwole and Taylor, 1987; Huey and Kingsolver, 1989). Temperature proved to be the factor most strongly influencing morning activity levels. Morning temperatures ranged from 18.5°C to 30.3°C. Afternoon temperatures were warmer (26.3–38.3°C) but with a similar range of ~12°C. Lizards are less active during cooler temperature periods, potentially explaining the higher influence of morning temperature on activity. Price-Rees *et al.* (2014) examined the activity patterns of blue-tongue lizards in the Australian wet–dry tropics and found that the peripheral temperature of the lizards correlated to ambient temperature as expected, but also that lizards would avoid extensive activity during the hottest part of the day. In semi-arid NSW, the hottest part of the day always fell within the afternoon period of activity; however, mean minutes moved per treatment remained similar for both time periods across the experimental period. Looking at temperature range for both time periods, we suggest there may be a potential temperature threshold for activity, but this may vary slightly for each lizard and may explain the lack of variation in activity totals between time periods. Further study in a similar setting is required to explain these results in more detail.

The physiological measures of body condition changed little throughout the experimental period. The Hb concentrations of all individuals were within the expected range for healthy *P. vitticeps* (Ellman, 1997; Mehren, 1999) indicating that, although living in challenging conditions, all lizards were relatively healthy (Smyth *et al.,* 2014; Arguedas *et al.,* 2018). Building on this, SBMI also indicated that all lizards were healthy. Similarly, there were no differences in body condition or growth of western fence lizards (*Sceloporus occidentalis*) subjected to repeated exposure of malathion, a ChE-inhibiting pesticide like fenitrothion (Holem *et al.,* 2008). However, Smyth *et al.* (2014) found that sleepy lizards (*Tiliqua rugosa)* from a baseline habitat (one that was relatively undisturbed with little agricultural modification) had better body condition than those from severely modified, fragmented habitat where various agricultural chemicals were used regularly. Our results are representative of a single-dose event, such as is common for locust control, and our body condition measures were not impacted by either pesticide. This differs to the widespread use of pesticides in agriculture, which represents a more repetitive, higher-dose exposure scenario, where body condition measures are more likely to be impacted (Relyea and Diecks, 2008; Smyth *et al.,* 2014). Sigouin *et al.* (2021) also highlights that, in some agricultural settings, species that inhabit those areas are most likely subject to long-term exposure at relatively low concentrations. Thus, we also suggest a physiological impact may be found during a longer monitoring period.

The lack of reptile ecotoxicology field studies may be a result of feasibility and difficulty in the nature of this research, or a lack of methodology and presence of a model species in such research (Sánchez-Bayo, 2011; Amaral *et al.,* 2012b). We have demonstrated in this study using novel, yet affordable technology, that activity and physiology measures of lizards in response to sublethal pesticide exposure can be accurately measured and done so in a model species representative of Reptilia. This research is a critical step forward in understanding non-target impacts of pesticides on reptiles around the world.

Our results suggest that the application of dietary intake of pesticides (here fipronil) by lizards has the potential to impact *P. vitticeps* populations caused by changes to normal activity patterns, further impacting survivability and reproductive behaviours, as well as their inability to depurate or detoxify pesticides within the body in the short term, posing latent toxicity risks. Further field-based research can build this case, along with progression to test molecular markers of stress, which may further reveal missed sublethal implications, and feeding with live, treated prey (Story *et al.,* 2021). We believe our study is highly applicable as a case study for not only arid-zone reptile species but given the immense lack of field-based and ecologically relevant experiments in ecotoxicology and conservation physiology-related research, our results form a base for future research in all reptile species as well.

## Funding

This work was supported by an ARC Linkage grant (LP160100686) awarded to the University of Wollongong, in collaboration with Macquarie University and the Australian Plague Locust Commission. Funding was also provided by the Ecological Society of Australia through the Holsworth Wildlife Research Endowment Grant.

## Data availability

The data underlying this article are available in this article and in the online supplementary material. Additional data will be made available on Mendeley Data repository if accepted for publication.

## Supplementary Material

Web_Material_coac024
