# Supplementary material for: Sublethal pesticide exposure influences behaviour, but not condition in a widespread Australian lizard
Source: Conserv Physiol. 2022 Apr 24;10(1):coac024. doi: 10.1093/conphys/coac024 (PMC9040277; doi:10.1093/conphys/coac024)
Supplement: Web_Material_coac024 [file web_material_coac024.docx]

Appendix S1.

**Sublethal pesticide exposure influences behaviour, but not condition in a widespread Australian Lizard.**

Isabella Contador-Kelsall, Kimberly Maute, Paul Story, Grant C Hose, Kristine French

**Methods for biomarkers of exposure: fenitrothion continued**

Following centrifugation, plasma was extracted and frozen at -20 °C after which samples were stored in a -80 °C freezer until analysis at the University of Canberra. Acetylcholinesterase activities were quantified from diluted plasma samples using methods described in Ellman et al. (1961) as modified by (1993) for use in a 96-well spectrophotometric plate reader (BioRad Benchmark Plus™, BioRad Laboratories CA, USA), equipped with software for enzyme kinetic analysis (BioRad Microplate Manager, BioRad Laboratories CA, USA). Assay reagents were obtained from Sigma-Aldrich Pty Ltd (Sydney, NSW, Australia).

All plasma samples were assayed in either duplicate or triplicate for total ChE and AChE activities, depending on the quantity of plasma available, at 25 °C for 2 mins (readings taken at 12 s intervals). Assay components were acetylthicholine iodide (AThCh, the ChE substrate, 5,5’-dithiobis(2-nitrobenzoic acid)(DTNB)), 0.05 M buffer (pH 7.4 and pH 8.0) and diluted enzyme (5-fold dilution) with a total volume of 250 µl per microplate well. The assay was initiated by the addition of AThCh to all other components. Acetylcholinesterase was differentiated from BChE by pre-incubation for 5 mins prior to the addition of AThCh with the specific BChE inhibitor, tetra-isopropyl pyrophosphoramide (iso-OMPA, 10^-6^ M). Butrylcholinesterase activity was calculated as the difference between TChE and AChE activities. Mouse serum (Sigma-Aldrich Pty Ltd, Sydney, NSW, Australia) was frozen in 0.5 mL aliquots and kept in a -80 °C freezer until used and provided a between-assay standard. Blank wells without added enzyme provided background colour formation. The increase in absorbance at 412 nm (∆A/min), corrected for blank, was converted to µmoles AThCh hydrolysed/min/mL of plasma using the molar extinction coefficient 13 600/cm/M (Ellman*, et al.*, 1961). Characterisation of enzyme activity for *P. vitticeps* was undertaken in previous research and the characterisation parameters can be found in Bain et al. (2004).

**Literature cited:**

**Bain D, Buttemer WA, Astheimer L, Fildes K, Hooper MJ** (2004) Effects of sublethal fenitrothion ingestion on cholinesterase inhibition, standard metabolism, thermal preference, and prey-capture ability in the australian central bearded dragon (pogona vitticeps, agamidae). *Environmental Toxicology and Chemistry* 23: 109-116

**Ellman GL, Courtney KD, Andres V, Featherstone RM** (1961) A new and rapid colorimetric determination of acetylcholinesterase activity. *Biochemical Pharmacology* 7: 88-95

**Gard NW, Hooper MJ** (1993) Age-dependent changes in plasma and brain cholinesterase activities of eastern bluebirds and european starlings. *Journal of Wildlife Diseases* 29: 1-7

**Figures**


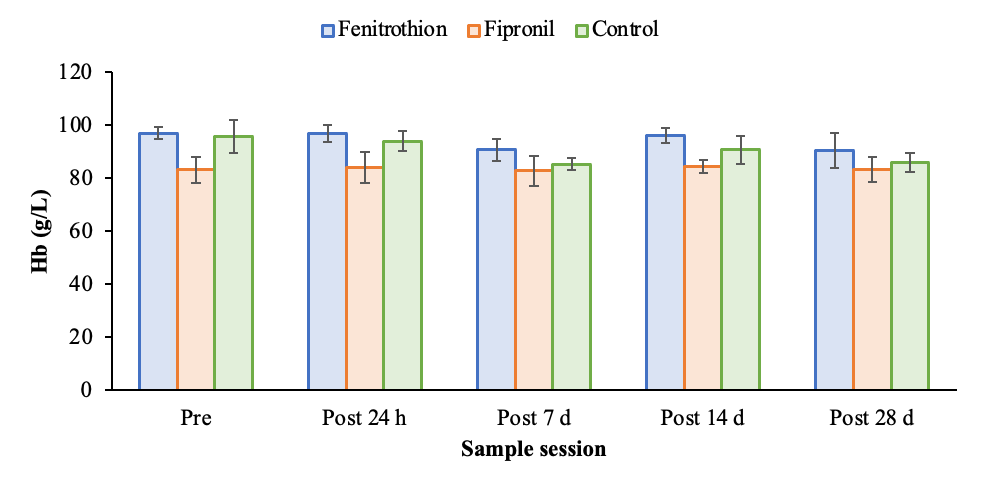


Figure. A1 Mean haemoglobin (Hb) for *Pogona vitticeps* in all treatments (fipronil, fenitrothion, control) at five time points. Hb data are shown as means with ± SE (n = 4-6 animals per treatment).


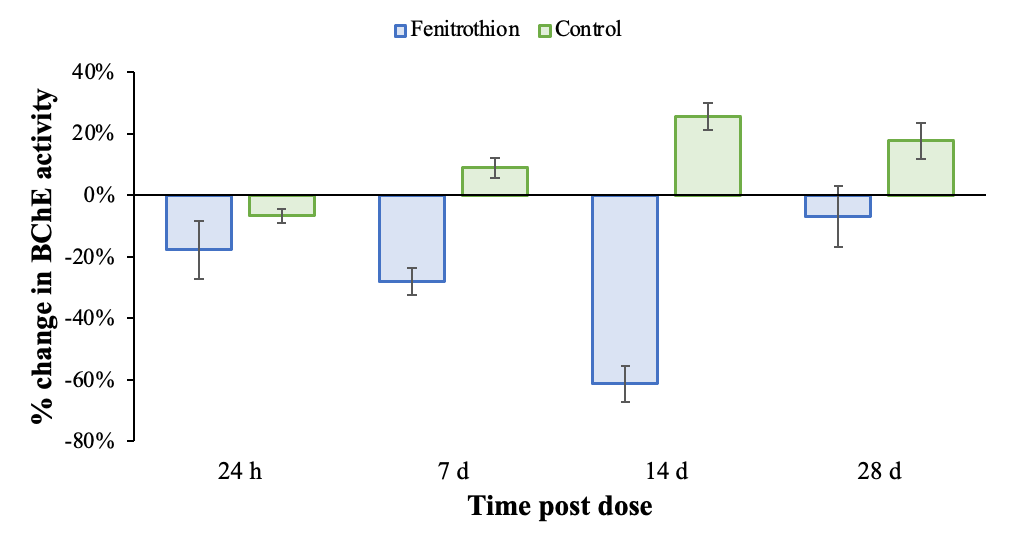


Figure. A2 Mean percentage change of butyrylcholinesterase (BChE) activity (µmol substrate/min/mL) in fenitrothion-dosed and control *Pogona vitticeps* (n = 4-6 animals per treatment) at four time points after being dosed. Data is displayed as means with ± SE.

Table A1. Post-Hoc Tukey HSD of scaled body mass index (SBMI) at all time periods across entire experimental period.

| **Sample session 1** | **Sample session 2** | **Mean difference** | **SE** | **P** |
| --- | --- | --- | --- | --- |
| Pre | Post 24 h | 1.781 | 16.177 | 1.000 |
|  | Post 7 d | -17.320 | 16.177 | 0.821 |
|  | Post 14 d | -9.681 | 16.177 | 0.975 |
|  | Post 28 d | -12.740 | 16.485 | 0.938 |
| Post 24 h | Pre | -1.781 | 16.177 | 1.000 |
|  | Post 7 d | **-19.100** | **16.177** | **0.762** |
|  | Post 14 d | -11.462 | 16.177 | 0.954 |
|  | Post 28 d | -14.520 | 16.485 | 0.903 |
| Post 7 d | Pre | 17.320 | 16.177 | 0.821 |
|  | Post 24 h | **19.100** | **16.177** | **0.762** |
|  | Post 14 d | 7.638 | 16.177 | 0.990 |
|  | Post 28 d | 4.580 | 16.485 | 0.999 |
| Post 14 d | Pre | 9.681 | 16.177 | 0.975 |
|  | Post 24 h | 11.462 | 16.177 | 0.954 |
|  | Post 7 d | -7.638 | 16.177 | 0.990 |
|  | Post 28 d | -3.058 | 16.485 | 1.000 |
| Post 28 d | Pre | 12.740 | 16.485 | 0.938 |
|  | Post 24 h | 14.520 | 16.485 | 0.903 |
|  | Post 7 d | -4.580 | 16.485 | 0.999 |
|  | Post 14 d | 3.058 | 16.485 | 1.000 |

*Notes:* Largest difference in bold. P-value (P), Standard error (SE).

Table A2. Post-Hoc Tukey HSD of Haemoglobin (Hb) at all time periods across entire experimental period.

| **Sample session 1** | **Sample session 2** | **Mean difference** | **SE** | **P** |
| --- | --- | --- | --- | --- |
| Pre | Post 24 h | -1.538 | 3.767 | 0.994 |
|  | Post 7 d | 7.182 | 3.941 | 0.370 |
|  | Post 14 d | 2.571 | 3.697 | 0.957 |
|  | Post 28 d | 6.923 | 3.767 | 0.362 |
| Post 24 h | Pre | 1.538 | 3.767 | 0.994 |
|  | Post 7 d | **8.720** | 4.007 | **0.203** |
|  | Post 14 d | 4.110 | 3.767 | 0.810 |
|  | Post 28 d | 8.462 | 3.836 | 0.192 |
| Post 7 d | Pre | -7.182 | 3.767 | 0.370 |
|  | Post 24 h | **-8.720** | 4.007 | **0.203** |
|  | Post 14 d | -4.610 | 3.941 | 0.768 |
|  | Post 28 d | -0.259 | 4.007 | 1.000 |
| Post 14 d | Pre | -2.571 | 3.697 | 0.957 |
|  | Post 24 h | -4.110 | 3.767 | 0.810 |
|  | Post 7 d | 4.610 | 3.941 | 0.768 |
|  | Post 28 d | 4.352 | 3.767 | 0.776 |
| Post 28 d | Pre | -6.923 | 3.767 | 0.362 |
|  | Post 24 h | -8.462 | 3.836 | 0.192 |
|  | Post 7 d | 0.259 | 4.007 | 1.000 |
|  | Post 14 d | -4.352 | 3.767 | 0.776 |

*Notes:* Largest difference in bold. P-value (P), Standard error (SE).

Table A3. Post-Hoc Tukey HSD of time of first movement at all time periods across entire experimental period.

| **Sample session 1** | **Sample session 2** | **Mean difference** | **SE** | **P** |
| --- | --- | --- | --- | --- |
| Pre | Post 24 h | 1:11:15.4 | 0:28:45.6 | 0.109 |
|  | Post 7 d | -1:45:27.5 | 0:28:45.6 | 0.005 |
|  | Post 14 d | -1:10:23.1 | 0:29:17.3 | 0.128 |
|  | Post 28 d | -0:15:04.6 | 0:29:53.5 | 0.987 |
| Post 24 h | Pre | -1:11:15.4 | 0:28:45.6 | 0.109 |
|  | Post 7 d | **-2:56:42.9** | 0:28:13.4 | **<0.0001** |
|  | Post 14 d | -2:21:38.5 | 0:28:45.6 | <0.0001 |
|  | Post 28 d | -1:26:20.0 | 0:20:22.6 | 0.036 |
| Post 7 d | Pre | 1:45:27.5 | 0:28:45.6 | 0.005 |
|  | Post 24 h | **2:56:42.9** | 0:28:13.4 | **<0.0001** |
|  | Post 14 d | 0:35:04.4 | 0:28:45.6 | 0.740 |
|  | Post 28 d | 1:30:22.9 | 0:29:22.5 | 0.025 |
| Post 14 d | Pre | 1:10:23.1 | 0:29:17.3 | 0.128 |
|  | Post 24 h | 2:21:38.5 | 0:28:45.6 | <0.0001 |
|  | Post 7 d | -0:35:04.4 | 0:28:45.6 | 0.740 |
|  | Post 28 d | 0:55:18.5 | 0:29:53.5 | 0.355 |
| Post 28 d | Pre | 0:15:04.6 | 0:29:53.5 | 0.987 |
|  | Post 24 h | 1:26:20.0 | 0:29:22.5 | 0.036 |
|  | Post 7 d | -1:30:22.9 | 0:29:22.5 | 0.025 |
|  | Post 14 d | -0:55.18.5 | 0:29:53.5 | 0.355 |

*Notes:* Significant effects in bold. P-value (P), Standard error (SE).

Table A4. Pearson’s correlation matrix for all time periods between temperature (AM) and fipronil sulfone (n=4).

| **Fipronil sulfone (µg/mL)** | **Temperature (°C)** |
| --- | --- |
| Pearson correlation | -0.255 |
| P | 0.278 |
| N | 20 |

*Notes:*. P-value (P), Number of cases (N).
